# Global regulator DksA modulates virulence of *Acinetobacter baumannii*

**DOI:** 10.1080/21505594.2021.1995253

**Published:** 2021-11-09

**Authors:** Nayeong Kim, Joo Hee Son, Kyeongmin Kim, Hyo Jeong Kim, Yoo Jeong Kim, Minsang Shin, Je Chul Lee

**Affiliations:** Department of Microbiology, School of Medicine, Kyungpook National University, Daegu, Republic of Korea

**Keywords:** *Acinetobacter baumannii*, DksA, virulence, quorum sensing system, biofilm formation

## Abstract

DksA with (p)ppGpp regulates a wide range of gene transcriptions during the stringent response. The aim of this study was to identify a DksA ortholog in *Acinetobacter baumannii* and clarify the roles of DksA in bacterial physiology and virulence. The ∆*dksA* mutant and its complemented strains were constructed using *A. baumannii* ATCC 17978. The *AlS_0248* in *A. baumannii* ATCC 17978 was identified to *dksA* using sequence homology, protein structure prediction, and gene expression patterns under different culture conditions. The ∆*dksA* mutant strain showed a filamentous morphology compared with the wild-type (WT) strain. Bacterial growth was decreased in the ∆*dksA* mutant strain under static conditions. Surface motility was decreased in the ∆*dksA* mutant strain compared with the WT strain. In contrast, biofilm formation was increased and biofilm-associated genes, such as *bfmR/S* and *csuC/D/E*, were upregulated in the ∆*dksA* mutant strain. The ∆*dksA* mutant strain produced less autoinducers than the WT strain. The expression of *abaI* and *abaR* was significantly decreased in the ∆*dksA* mutant strain. Furthermore, the ∆*dksA* mutant strain showed less bacterial burden and milder histopathological changes in the lungs of mice than the WT strain. Mice survival was also significantly different between the ∆*dksA* mutant and WT strains. Conclusively, DksA is directly or indirectly involved in regulating a wide range of genes associated with bacterial physiology and virulence, which contributes to the pathogenesis of *A. baumannii*. Thus, DksA is a potential anti-virulence target for *A. baumannii* infection.

## Introduction

*Acinetobacter baumannii* is an important opportunistic pathogen that causes a hospital-acquired infection in severely ill patients [1,2]. Current therapeutic options for *A. baumannii* infections are limited due to its antimicrobial resistance, posing a significant threat to global public health [3,4]. The World Health Organization declared carbapenem-resistant *A. baumannii* as a top priority pathogen for investing in new therapeutic drugs [5]. *A. baumannii* is considered to be a low virulent pathogen [1,6], but several virulence factors, including outer membrane proteins [7–9], phospholipases [10], lipopolysaccharides [11], protein secretion systems [12], capsular polysaccharides [13], and the acquisition and utilization of metal ions [14,15], have been identified in this microorganism. The ability to survive and persist in harsh environments or stressful conditions is also responsible for the pathogenicity of *A. baumannii* [1,16]. Two *A. baumannii* strains that showed the same pulsotype but produced different levels of exopolysaccharides exhibited different clinical scores and mortalities in a mouse pneumonia model [17]. This suggests that different regulation of virulence factors directly contributes to clinical outcomes. Understanding global regulatory mechanisms of virulence factors is required to identify new therapeutic targets for drug-resistant *A. baumannii* infections.

The stringent response is a global regulatory mechanism of genes that adapts bacteria to many stress conditions [18,19]. The production of signaling molecules guanosine-5´,3´-pentaphosphate (pppGpp) and guanosine-5´,3´-tetraphosphate (ppGpp), collectively known as (p)ppGpp, is the hallmark of this response [19,20]. (p)ppGpp interacts with two binding sites of RNA polymerase (RNAP), which is either activated or repressed in a promoter-dependent way, changing the expression of genes in response to various environmental stresses [21–23]. Binding of (p)ppGpp to the interface of ω and β′ subunits induces a conformational change of RNAP and influences the lifetime of the open complex [22,23]. Binding of (p)ppGpp to the interface of the secondary channel of β′ subunits influences the transcription initiation [23]. The DnaK suppressor protein (DksA), an RNAP-binding transcription factor of the stringent response, binds to the secondary channel of RNAP, which modulates the RNAP activity [24,25]. In *Escherichia coli*, (p)ppGpp and DksA function synergistically to downregulate the transcription of genes associated with the synthesis of translational machinery and upregulate genes associated with the stress tolerance during the stringent response [24,26]. In addition to coordinated gene regulation, (p)ppGpp and DksA regulate gene expression independently or sometimes conduct an opposing role [27]. The small cytosolic protein DksA controls various cellular physiological processes, including amino acid biosynthesis [26], translation [24], DNA repair [28,29], cellular division [30], and oxidative stress responses [31] in Gram-negative bacteria. DksA has been reported to regulate virulence and antimicrobial resistance in Gram-negative pathogens [32–35]. Thus, DksA may work independently or along with (p)ppGpp to affect the RNAP activity, contributing to bacterial physiology, pathogenesis, and antimicrobial resistance.

In *A. baumannii*, RelA, a (p)ppGpp synthetase, has been recently characterized. ∆*relA* mutant exhibits hypermotility, cell elongation, and low virulence in the *Galleria mellonella* infection model [36]. Furthermore, (p)ppGpp-deficient mutants are more susceptible to antimicrobial agents, including gentamicin, tetracycline, erythromycin, and trimethoprim, than wild-type (WT) *A. baumannii* ATCC 17978 strain by downregulating efflux pump genes [37]. Although the functional characterization of (p)ppGpp in *A. baumannii* has been made, DksA has not been identified. Therefore, this study identified a DksA ortholog in *A. baumannii* and clarified the roles of DksA in bacterial physiology and virulence using WT *A. baumannii* ATCC 17978, ∆*dksA* mutant, and *dksA*-complemented strains.

## Materials and methods

### Bacterial strains

Bacterial strains and plasmids used in this study are listed in Supplementary Table S1. *A. baumannii* and *E. coli* strains were grown in lysogeny broth (LB) at 37°C. To select colonies during the construction of mutant and complementary strains, antimicrobial agents, chloramphenicol (20 μg/ml) or kanamycin (50 μg/ml), were added to the growth medium.

### Sequence homology and prediction of protein structure

Nucleotide sequences of *A1S_0248* were obtained from the National Center for Biotechnology Information (NCBI) (https://www.ncbi.nlm.nih.gov/). Multiple alignments of the translated sequences of *A1S_0248* with DksA of other bacterial species were performed using the constraint-based alignment tool for multiple protein sequences (COBALT) (https://www.ncbi.nlm.nih.gov/tools/cobalt/re_cobalt.cgi). WebLogo 3 was used to identify the sequence logo between *E. coli* DksA and the translated sequences of *A1S_0248* of *A. baumannii* ATCC 17978 (http://weblogo.threeplusone.com/). The three-dimensional protein structure of the *A1S_0248* gene product was predicted using SWISS-MODEL (https://swissmodel.expasy.org/).

### Construction of the ∆dksA mutant and dksA-complemented strains

The ∆*A1S_0248* mutant strain (KM0248D) was constructed using *A. baumannii* ATCC 17978 by a markerless gene deletion method as previously described [38]. The complementation of *A1S_0248* in the KM0248D strain was conducted using an overlap extension polymerase chain reaction (PCR) and the *A1S_0248*-complemented strain (KM0248C) was constructed. The specific PCR primers used to construct the mutant and complemented strains are listed in Supplementary Table S2. The construction of mutant and complemented strains is presented in Supplementary Figure S1 and the Supplementary Materials and methods section in detail.

### Bacterial growth curve and gram staining

*A. baumannii* strains were cultured overnight in LB and then diluted to an optical density at 600 nm (OD_600_) of 1.0. The bacterial suspension was diluted at 1:20 in fresh LB and cultured under shaking and static conditions for 24 and 48 h at 37°C, respectively. Bacterial growth was measured at OD_600_ using a spectrophotometer (Biochrom, Cambridge, UK). A bacterial growth assay was performed in two independent experiments. *A. baumannii* strains were cultured on M9 agar plates to determine the growth of bacteria in minimal media. For Gram staining, bacteria were grown in LB under shaking conditions to reach the indicated OD_600_. Bacterial samples were stained by Gram’s reagents and observed using the light microscope (Eclipse E600, Nikon, Tokyo, Japan).

### RNA isolation and quantitative real-time PCR (qPCR)

*A. baumannii* ATCC 17978 was cultured in LB under static or shaking conditions at 23, 30, or 37°C for 18 h to analyze the expression of *dksA* under different culture conditions. *A. baumannii* strains were cultured in LB under static or shaking conditions for 18 or 24 h to analyze the expression of virulence-associated genes. Total RNA was isolated from *A. baumannii* strains using the RNeasy Mini Kit (Qiagen, Valencia, CA, USA). Total RNA (1.5 μg) was reverse-transcribed to synthesize cDNA using random hexamer primers and TOPscript^TM^ reverse transcriptase (Enzynomics, Daejeon, Korea). The specific primers for target genes are listed in Supplementary Table S3. Gene transcripts were quantified using TOPreal™ qPCR 2Χ PreMIX (SYBR Green with high ROX) (Enzynomics) with a StepOnePlus^TM^ Real-time PCR Systems (Applied Biosystems, Foster City, CA, USA). The fold change in the expression level of each gene was calculated by the ∆∆Ct method using the 16S rRNA gene as an internal control. The expression of genes was performed in two or three independent experiments

### Surface motility assay

*A. baumannii* strains were cultured in LB under shaking conditions overnight. Bacterial cultures were diluted in LB to an OD_600_ of 1.0, and bacterial suspension (2 μl) was inoculated onto motility agar plates containing tryptone 1%, yeast extract 0.5%, and Eiken agar powder 0.3% [39]. The diameters of bacterial migration were measured at 4, 7, and 12 h. The surface motility assay was performed in three independent experiments.

### Biofilm formation assay

For biofilm formation assay of *A. baumannii* strains, LB broth without sodium chloride was used. Bacteria were cultured overnight in LB and then diluted to an OD_600_ of 1.0. The bacterial suspension (2 ml) diluted with 1:40 in fresh LB was inoculated into wells of a 24-well plate and incubated without shaking for 10, 24, and 48 h. After removing liquid media containing the planktonic cells, biofilm cells were washed with phosphate-buffered saline (PBS) and stained with a 0.1% (w/v) crystal violet solution for 15 min. The biofilm cell-associated dye was eluted with 95% ethanol and absorbance of the eluted solvent was measured at OD_570_. Biofilm mass at OD_570_ was normalized to bacterial growth at OD_600_. The assay was performed in three independent experiments.

### Bioassay for detecting quorum sensing molecules

The reporter strain *Agrobacterium tumefaciens* NT1 (pDCI41E33) was cultured in the defined minimal medium at 30°C [40]. *A. baumannii* strains were cultured in LB overnight at 37°C and then adjusted to an OD_600_ of 1.0. Bacterial samples (5 μl) were spotted onto the bioassay plate overlaid with the reporter strain [41]. As a control, *N*-(3-hydroxy-dodecanoyl)-DL-homoserine lactone (Sigma-Aldrich, St. Louis, MO, USA) was used. The blue zone was measured after the plate was incubated at 30°C for 18 h. The bioassay was performed in three independent experiments.

### Animal experiments

Female BALB/c mice (eight-week-old) were kept under conventional conditions. Cyclophosphamide was used to induce neutropenia in mice as previously described [42]. Neutropenic mice were intraperitoneally infected with 5 × 10^8^ colony forming units (CFUs) of the *A. baumannii* strains, and the survival of mice was evaluated for 5 days after bacterial infection. Neutropenic mice were infected with 5 × 10^7^ CFUs of the *A. baumannii* strains intratracheally to evaluate bacterial loads and histological changes in the lungs. The surviving mice were sacrificed 24 h post-infection; also, cardiac puncture was performed to obtain blood samples, and both lungs were removed. The CFUs in blood and right lung homogenates were determined on LB agar plates. Left lung tissues were fixed in formalin solution and stained with hematoxylin and eosin. All animal experiments were performed in accordance with the Institutional Animal Care and Use Committee of Kyungpook National University.

### Statistical analysis

Data were analyzed by one-way analysis of variance with Dunnett’s post hoc analysis and Student’s t-tests using GraphPad Prism 5.0 (San Diego, CA, USA). Differences at *p* < 0.05 were considered statistically significant.

## Results

### Identification of dksA in A. baumannii

**Figure 1.**
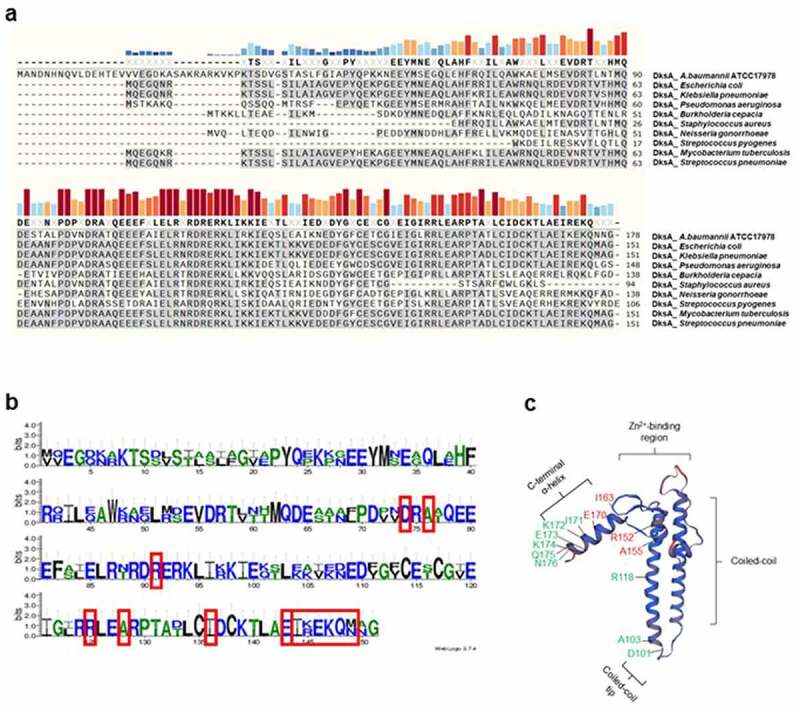
Identification of DksA in *A. baumannii*. (a) Multiple alignments of translated sequences of *A1S_0248* with DksA of other bacterial species. The gray highlight indicates identical amino acids with *E. coli* DksA. The bold characters indicate >50% consensus amino acids among the tested bacterial species. Sequence conservation is presented as the height of colored bars. The accession number of DksA proteins is ABO10723 in *A. baumannii* ATCC 17978, VWQ00927 in *E. coli*, CDO15944 in *Klebsiella pneumoniae*, WP_058139749 in *Pseudomonas aeruginosa*, KIS47657 in *Burkholderia cepacia*, RTY79203 in *Staphylococcus aureus*, WP_047919748 in *Neisseria gonorrheae*, WP_148842572 in *Streptococcus pyogenes*, SGC70216 in *Mycobacterium tuberculosis*, and VTQ30573 in *Streptococcus pneumoniae*. (b) Sequence logo of DksA between *E. coli* and *A. baumannii* ATCC 17978 using the WebLogo 3. The red boxes are essential amino acids for RNAP binding and DksA activity in *E. coli*. The number is based on the amino acid sequences of *E. coli* DksA. Furthermore, 151 amino acids of *E. coli* DksA (the first number in parenthesis) are compared with the translated amino acid sequences of *A1S_0248* of *A. baumannii* ATCC 17978 (the second number in parenthesis) in the boxes as follows: D (74, 101); A (76, 103); R (91, 118); R (125, 152); A (128, 155); I (136, 163); E (143, 170); I-M (144–149, 171–176). (c) The predicted three-dimensional structure of proteins encoded by *A1S_0248* using Swiss-MODEL. Red and green colors indicate the amino acids for the RNAP binding and DksA activity, respectively

**Figure 2.**
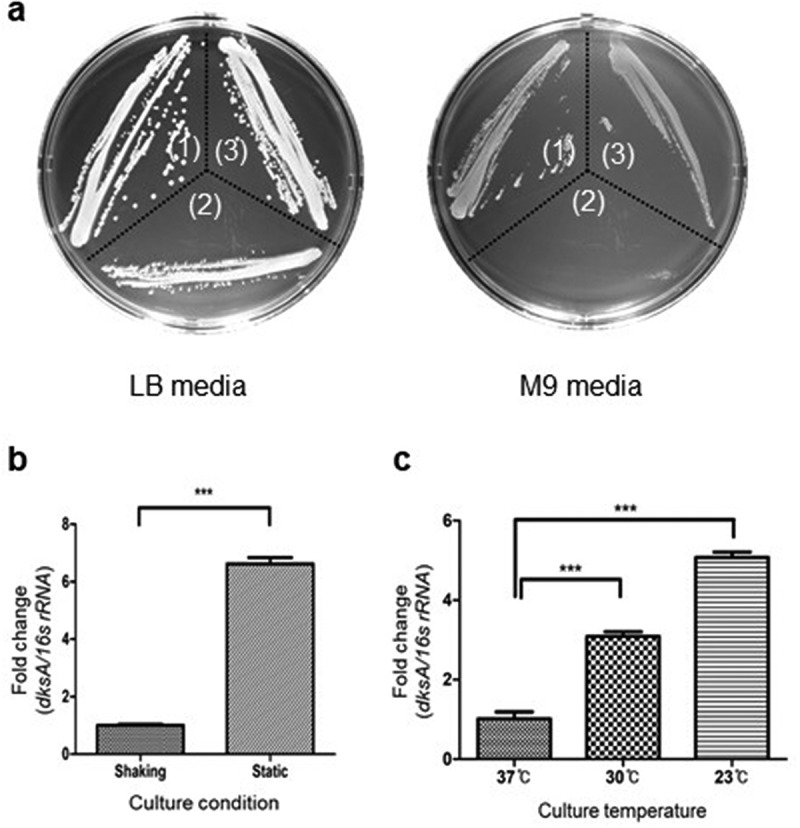
Growth of *A. baumannii* strains in minimal media and the expression of *dksA* in *A. baumannii* ATCC 17978 under different culture conditions. (a) WT *A. baumannii* ATCC 17978 (1), ∆*dksA* mutant (2), and *dksA*-complemented (3) strains were grown on LB and M9 agar plates for 24 h. (b and c) The expression of *dksA* in *A. baumannii* ATCC 17978 was analyzed using qPCR. (b) Bacteria were cultured in LB under shaking or static conditions for 18 h. (c) Bacteria were cultured in LB under shaking at 23, 30, or 37°C for 18 h. The data are presented as the mean ± SD of three independent experiments. *** *p* < 0.001

The *A1S_0248* gene (537 bases) of *A. baumannii* ATCC 17978 was predicted to be *dksA* at NCBI, but the protein encoded by *A1S_0248* had not been characterized. The homology of translated sequences of *A1S_0248* ranged from 28.1% in the DksA of *Streptococcus pyogenes* (106 amino acids) to 51.7% in the DksA of *E. coli* (151 amino acids) (Figure 1(a)). The sequence logo showed that all sequences required for RNAP binding and DksA activity were identical, except for R145 and M149 in *E. coli* DksA, between the *A1S_0248* gene product and *E. coli* DksA [25] (Figure 1(b)). The protein structure of *A1S_0248* obtained using the SWISS-MODEL showed a high structural similarity with *E. coli* DksA [23] (Figure 1(c)). Next, to determine whether *A1S_0248* was responsible for the stringent response, *A. baumannii* ATCC 17978, ∆*A1S_0248* mutant, and *A1S_0248*-complemented strains were cultured on M9 minimal media. WT and *A1S_0248*-complemented strains were grown on LB and M9 agar plates, but the ∆*A1S_0248* mutant exhibited no growth on M9 minimal media (Figure 2(a)). *A. baumannii* ATCC 17978 was cultured under different culture conditions, and the expression of *A1S_0248* was analyzed using qPCR. The expression of *A1S_0248* was upregulated more than 6-folds in bacteria cultured under static conditions than shaking conditions (Figure 2(b)). The expression of *A1S_0248* was higher in bacteria cultured at 23°C (Figure 2(c)). Based on the sequence homology, protein structure prediction, bacterial growth on minimal media, and gene expression patterns under different culture conditions, *A1S_0248* will hereafter be referred to as *dksA*.

### Construction of ∆dksA mutant and dksA-complemented strains

To investigate the roles of *A. baumannii* DksA in the bacterial physiology and virulence, ∆*dksA* mutant (KM0248D) and *dksA*-complemented (KM0248C) strains were constructed using *A. baumannii* ATCC 17978 (Supplementary Figure S1(a) and S1(b)). PCR analysis confirmed the deletion and complementation of *dksA* (Supplementary Figure S1(c)). qPCR was performed to analyze the expression of *dksA* in WT, ∆*dksA* mutant, and complemented strains. The expression of *dksA* was observed in WT and *dksA*-complemented strains but not in ∆*dksA* mutant strain (Supplementary Figure S1(d)).

### Differences in cell morphology and growth kinetics between WT and ∆dksA mutant strains

**Figure 3.**
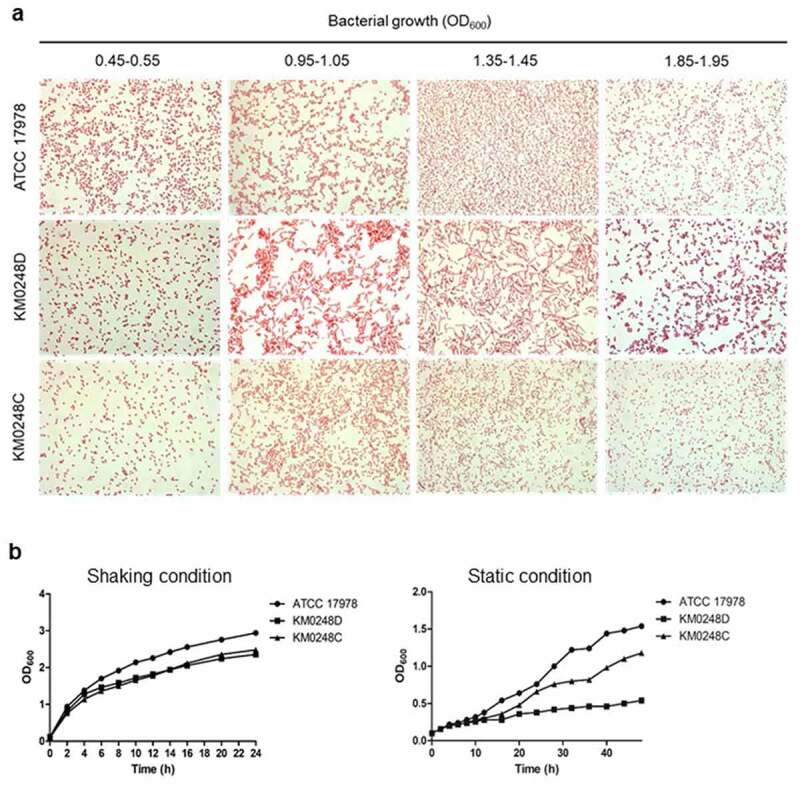
Cellular morphology and growth kinetics of *A. baumannii* strains. (a) WT, ∆*dksA* mutant (KM0248D), and *dksA*-complemented (KM0248C) strains were cultured in LB under shaking conditions to reach the indicated OD_600_ and stained by Gram staining. Bacterial morphology was observed using a light microscope. Magnification, 1,000X. (b) *A. baumannii* strains were cultured in LB under shaking conditions for 24 h and LB under static conditions for 48 h. Bacterial growth was measured at OD_600_. The result is representative of two independent experiments

To assess whether the deletion of *dksA* could affect bacterial morphology, such as (p)ppGpp-deficient mutant [36,37], *A. baumannii* strains were cultured in LB to reach the indicated OD_600_, and bacterial morphology was examined by Gram staining. The ∆*dksA* mutant showed more filamentous morphology than WT and *dksA*-complemented strains at an OD_600_ of 0.95–1.05 and 1.35–1.45. In contrast, the ∆*dksA* mutant showed similar morphology at OD_600_ of 1.85–1.95 compared with WT and *dksA*-complemented strains (Figure 3(a)). To determine whether the deletion of *dksA* could affect growth of *A. baumannii*, bacteria were cultured in LB under static or shaking conditions, and bacterial growth was measured at OD_600_. Bacterial growth was similar among the WT, ∆*dksA* mutant, and *dksA*-complemented strains under shaking conditions, although the WT strain exhibited a higher OD_600_ than ∆*dksA* mutant and *dksA*-complemented strains (Figure 3(b)). However, a significant growth retardation of ∆*dksA* mutant under static conditions was observed, although WT and *dksA*-complemented strains showed different growth rates (Figure 3(b)). These results suggest that the deletion of *dksA* alters cellular morphology and growth kinetics under static conditions in *A. baumannii* ATCC 17978.

### Differences in surface motility and biofilm formation between WT and ∆dksA mutant strains

**Figure 4.**
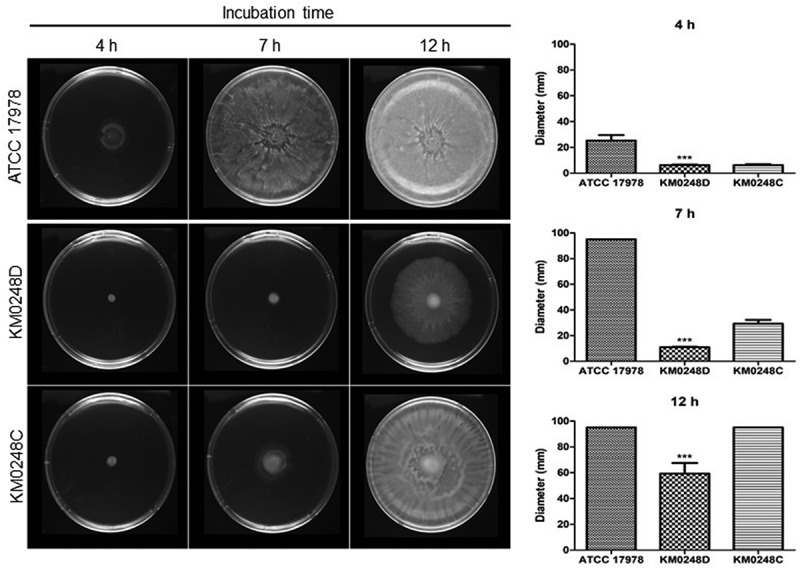
Surface motility of *A. baumannii* strains. WT *A. baumannii* ATCC 17978, ∆*dksA* mutant (KM0248D), and *dksA*-complemented (KM0248C) strains were cultured in motility agar plates for 4, 7, and 12 h, and the diameters (mm) of bacterial migration on agar plates were measured. Data are presented as the mean ± SD of three independent experiments. *** *p* < 0.001 compared with the WT strain

**Figure 5.**
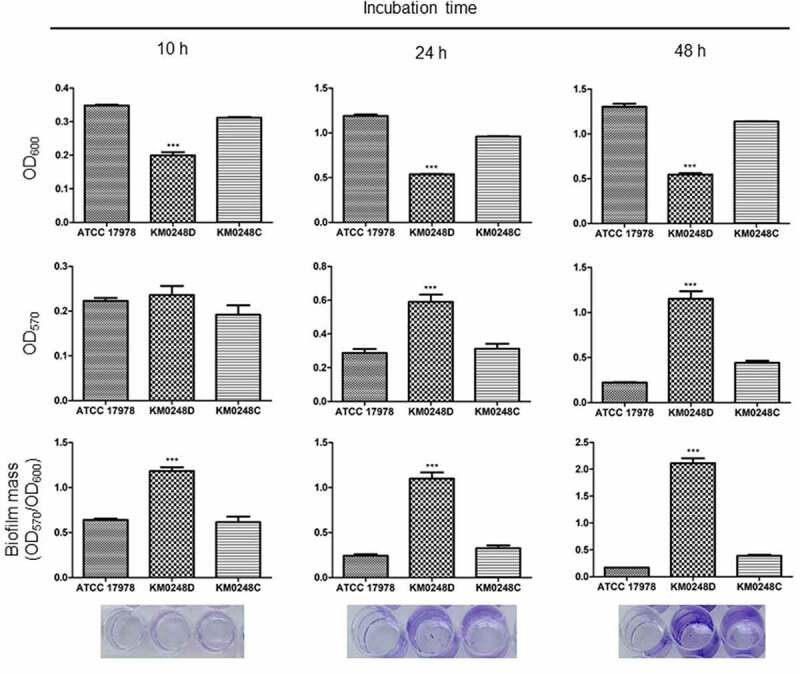
Biofilm formation of *A. baumannii* strains. WT *A. baumannii* ATCC 17978, ∆*dksA* mutant (KM0248D), and *dksA*-complemented (KM0248C) strains were cultured in 24-well plates, and biofilm mass was stained with crystal violet. Total bacterial growth and biofilms were measured at OD_600_ and OD_570_, respectively, and the biofilm mass was analyzed by OD_570_/OD_600_. The data are presented as the mean ± SD of three independent experiments. The pictures were biofilms stained with crystal violet. *** *p* < 0.001 compared with the WT strain

**Figure 6.**
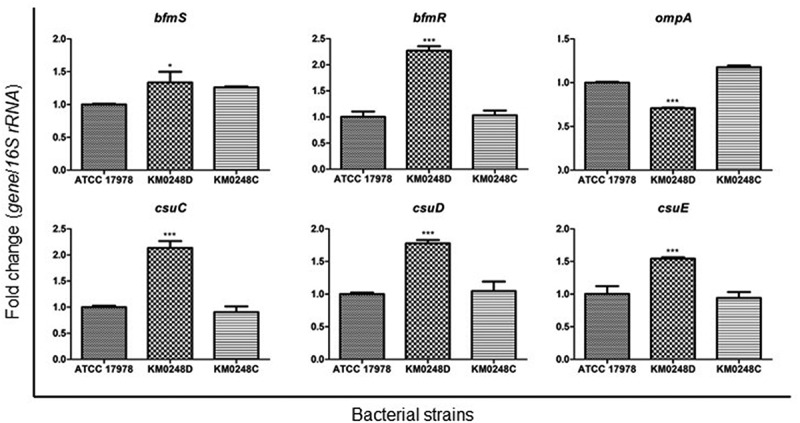
Expression of genes associated with biofilm formation in *A. baumannii* strains. WT *A. baumannii* ATCC 17978, ∆*dksA* mutant (KM0248D), and *dksA*-complemented (KM0248C) strains were cultured in LB under static conditions for 24 h. The expression of genes was analyzed using qPCR. The data are the expression levels of mean ± SD of target genes in each strain relative to the expression of these genes in the WT strain. The experiments were performed three times independently. * *p* < 0.05, *** *p* < 0.001 compared with the WT strain

To determine whether the deletion of *dksA* could affect surface motility of *A. baumannii*, bacteria were cultured in the motility agar plates, and migration of bacteria into the agar plates was measured. The ∆*dksA* mutant strain exhibited significantly smaller migration diameters than the WT strain at all tested times (Figure 4). The surface motility of *dksA*-complemented strain was partially restored at 7 h, but was fully restored at 12 h. Next, to assess whether the deletion of *dksA* could affect biofilm formation of *A. baumannii*, bacteria were cultured in 24-well plates for 48 h, and biofilm mass was measured. In contrast to the surface motility, ∆*dksA* mutant produced significantly more biofilm mass than WT and *dksA*-complemented strains (Figure 5). The difference in biofilm mass between WT and ∆*dksA* mutant strains was prominent at 48 h. To understand the regulation of biofilm-associated genes by DksA, the expression of *bfmR/S* for the two-component system, *csuC/D/E* for the chaperone-usher pilus system, and *ompA* for outer membrane protein A was analyzed in WT, ∆*dksA* mutant, and *dksA*-complemented strains cultured under static conditions for 24 h using qPCR. The expression of *bfmR/S* and *csuC/D/E* was significantly upregulated in the ∆*dksA* mutant strain, whereas the *ompA* expression was significantly downregulated in the ∆*dksA* mutant strain (Figure 6). These results suggest that DksA modulates surface motility and biofilm formation of *A. baumannii* ATCC 17978.

### Differences in the production of autoinducers between WT and ∆dksA mutant strains

**Figure 7.**
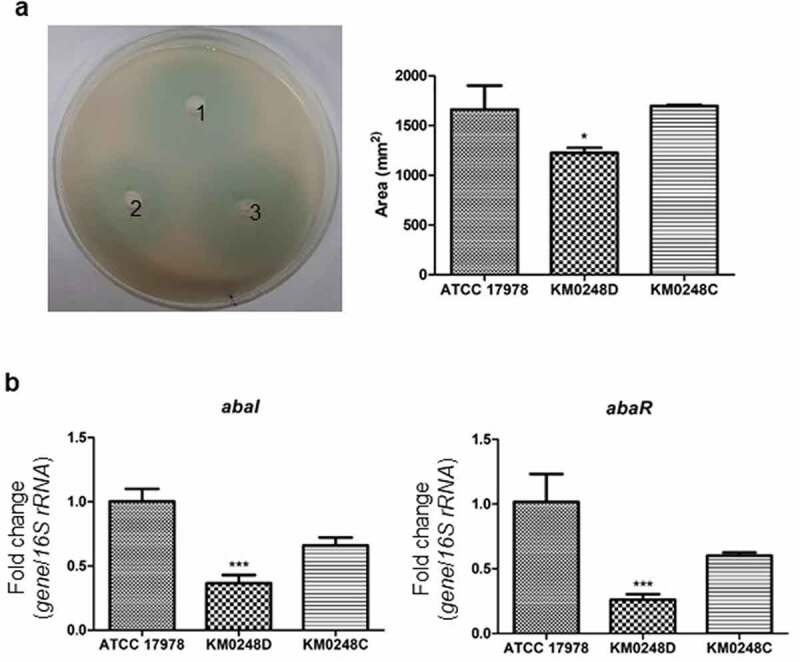
Production of autoinducers and expression of *abaI* and *abaR* in *A. baumannii* strains. (a) WT *A. baumannii* ATCC 17978 (1), ∆*dksA* mutant (KM0248D, 2), and *dksA*-complemented (KM0248C, 3) strains were cultured in the bioassay agar plates overlaid with *A. tumefaciens* (pDCI41E33) for 18 h, and blue zone was measured. The experiments were performed three times independently. * *p* < 0.05 compared with the WT strain. (b) *A. baumannii* strains were cultured in LB under static conditions for 18 h. qPCR was performed to analyze the expression of genes. The data are presented as the mean ± SD of three independent experiments. *** *p* < 0.001 compared with the WT strain

To determine whether the deletion of *dksA* could affect the quorum sensing systems of *A. baumannii*, three *A. baumannii* strains were spotted onto plates overlaid with the reporter strain, and autoinducer production was determined. The ∆*dksA* mutant produced a smaller blue zone than WT and *dksA*-complemented strains (Figure 7(a)). The expression of *abaI* and *abaR* was significantly decreased in the ∆*dksA* mutant than the WT strain (Figure 7(b)). These results suggest that DksA regulates quorum sensing systems in *A. baumannii* ATCC 17978.

### Effect of DksA on mice infected with A. baumannii strains

**Figure 8.**
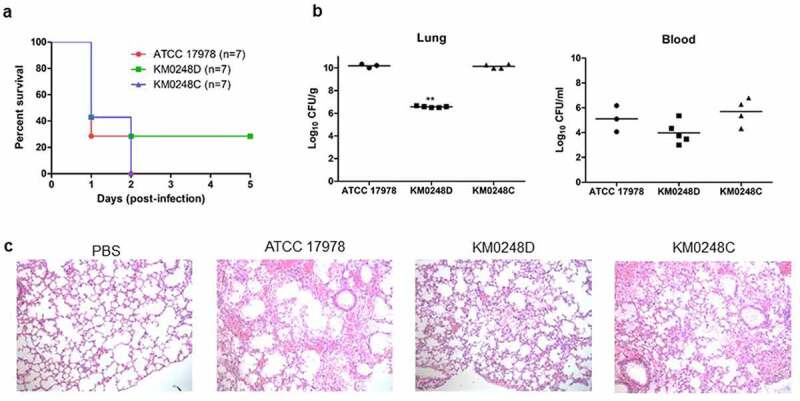
Survival rates and pathologies in mice infected with WT *A. baumannii* ATCC 17978, ∆*dksA* mutant (KM0248D), and *dksA*-complemented (KM0248C) strains. (a) Neutropenic mice were infected with 5 × 10^8^ CFUs of *A. baumannii* strains intraperitoneally. The survival of mice was determined for five days post-infection. (b and c) Seven neutropenic mice in each group were infected with 5 × 10^7^ CFUs of *A. baumannii* strains intratracheally. The surviving mice were sacrificed 24 h post-infection. (b) CFUs in the right lung and blood were determined. ** *p* < 0.01 compared with the WT strain. (c) Left lung tissue was stained with hematoxylin and eosin. Magnification, 100X

Neutropenic mice were intraperitoneally infected with *A. baumannii* strains, and their survival was monitored for five days. All mice infected with WT and *dksA*-complemented strains died within two days, whereas two (28.6%) of seven mice infected with the ∆*dksA* mutant survived (Figure 8(a)). Seven neutropenic mice in each group were infected with *A. baumannii* strains intratracheally to assess whether deleting *dksA* affects the pathogenesis of *A. baumannii*. Bacterial burden in the lungs and blood of surviving mice was determined 24 h after bacterial injection. Three, five, and four mice infected with WT, ∆*dksA* mutant, and *dksA*-complemented strains survived at 24 h post-infection, respectively. The CFUs in the lungs showed a difference of >1,000-folds between WT and ∆*dksA* mutant strains (Figure 8(b)). The mean value of CFUs in the blood was slightly decreased in the mice infected with the ∆*dksA* mutant strain, but significant difference was not observed between the two groups. WT and *dksA*-complemented strains induced severe lung pathologies, such as destruction of alveoli, congestion, and exudates, whereas mild pathological events were observed in the lungs of mice infected with the ∆*dksA* mutant strain (Figure 8(c)). These results suggest that DksA directly contributes to the pathogenesis of *A. baumannii* ATCC 17978 *in vivo*.

## Discussion

The present study demonstrates that *A. baumannii* DksA plays a role in bacterial morphology and growth under static conditions. Furthermore, *A. baumannii* DksA modulates biofilm formation, surface motility, and production of autoinducers *in vitro* and contributes to the pathogenesis *in vivo*. Overall, *A. baumannii* DksA plays multifaceted roles in bacterial physiology and virulence.

We first tried identifying a DksA ortholog in *A. baumannii*. The *A1S_0248* gene in *A. baumannii* ATCC 17978 was predicted to be *dksA* at NCBI. The translated sequences of *A1S_0248* were conserved among the predicted *dksA* of the sequenced *A. baumannii* strains (data not shown) but were different from *E. coli* DksA in size and amino acid sequences. In *E. coli* DksA, R125, A128, I136, and E143 and I144-M149 are critical residues interacting with the β′ rim helices and βSI1 of RNAP, respectively [25]. D74, A76, and R91 are essential residues for DksA activity [25]. These amino acids essential for RNAP binding and DksA activity were conserved in the *A1S_0248* gene products, except for K145 and N149. Additionally, structural analysis predicted that *A1S_0248*-encoded proteins carried zinc-binding, coiled-coil, and C-terminal helix domains. The synthesis of (p)ppGpp and DksA may be altered in response to the stress conditions [32,43]. In this study, *A. baumannii* ATCC 17978 strain expressed more *dksA* in harsh culture conditions, such as static conditions and low culture temperature, than in shaking conditions and optimal culture temperature. Mason *et al*. [32] also demonstrated that the expression of *dksA* was regulated by different environmental conditions, such as growth phase, growth temperature, and pH in *Borrelia burgdorferi*. These results suggest that *A1S_0248* in *A. baumannii* ATCC 17978 encodes a DksA.

Herein, we demonstrated that ∆*dksA* mutants of *A. baumannii* ATCC 17978 exhibited a filamentous morphology during the specific growth phases (OD_600_ of 0.95–1.45). Moreover, this mutant strain exhibited growth retardation under stationary conditions but not under shaking conditions. In a previous study, (p)ppGpp-deficient mutants also exhibited more filamentous morphology than the WT *A. baumannii* ATCC 17978 [37]. The (p)ppGpp-deficient mutants of *A. baumannii* ATCC 17978 did not show any growth retardation under shaking conditions. However, the growth of (p)ppGpp-deficient mutants under static conditions had not been determined. In *E. coli*, (p)ppGpp-deficient and ∆*dksA* mutants exhibit more filamentous morphology than the WT strain [27]. (p)ppGpp and DksA work together to modulate bacterial growth and viability [30,44]. Although the exact mechanisms explaining the differences in bacterial morphology and growth between WT and ∆*dksA* mutant strains of *A. baumannii* were not determined in this study, (p)ppGpp and DksA may regulate genes associated with cell division or morphogenesis in the same manner.

This study demonstrated that DksA plays an opposite role in biofilm formation and surface motility of *A. baumannii* ATCC 17978. ∆*dksA* mutant strains produced a higher biofilm mass than the WT strain, whereas ∆*dksA* mutants were less motile than the WT strain. The role of (p)ppGpp in biofilm formation was different among bacterial species: (p)ppGpp-deficient mutants of *E. coli* and *Pseudomonas putida* formed increased biofilms in LB medium [45,46], whereas (p)ppGpp-deficient mutants of *Enterococcus faecalis* and *Vibrio cholerae* formed decreased biofilms [47,48]. As observed in the growth kinetics of *A. baumannii* strains under static conditions, the ∆*dksA* mutant exhibited growth retardation at all three tested times in the biofilm assay conditions. The high ability to produce biofilm mass (OD_570_/OD_600_) of ∆*dksA* mutants at 10 h of incubation was due to the growth retardation of this mutant strain. However, biofilm mass (OD_570_) of the ∆*dksA* mutant was significantly increased at 24 and 48 h compared with the WT strain, although the growth (OD_600_) of the ∆*dksA* mutant was decreased at these times. Genes associated with biofilm formation, such as *bfmR/S* and *csu* operon, were upregulated in the ∆*dksA* mutants, whereas *ompA* was downregulated in the ∆*dksA* mutants. In contrast to biofilm formation, the WT strain exhibited more motility than the ∆*dksA* mutant. The (p)ppGpp-deficient and ∆*dksA* mutant strains were less motile than the WT *E. coli* strain [27]. However, the ∆*relA* mutant strains of *A. baumannii* showed hypermotility in motility agar plates [36]. These results suggest that (p)ppGpp and DksA play an opposite role in the surface motility of *A. baumannii*. Genetic analysis of *A. baumannii* identified 30 genes that were associated with surface motility, including genes encoding the 1,3-diaminopropane biosynthesis, alarmone/stress metabolism, DNA modification/repair/uptake, lipopeptide synthesis/export, outer membrane proteins, oxidative stress, purine/pyrimidine/folate biosynthesis, RNA modification/regulation, and others [49]. Of the genes associated with surface motility, *ompA* was downregulated in the ∆*dksA* mutants. However, the expression of *ddc* and *dat* for 1,3-diaminopropane biosynthesis was upregulated in the ∆*dksA* mutants (Supplementary Figure S2). Altogether, these results suggest that DksA negatively regulates the expression of *bfmR/S* and *csu* operon, which modulates the biofilm formation in *A. baumannii*. However, regulation of genes associated with surface motility of *A. baumannii* by DksA should be further investigated.

In *A. baumannii*, 3-hydroxy-dodecanoyl-_L_-homoserine lactone is a major quorum sensing molecule [50]. The autoinducers produced by the LuxI-type synthase AbaI bind to their corresponding transcriptional activator AbaR. These complex bind to lux boxes and then activates the transcription of target genes. AbaR is required for expressing *abaI* and producing autoinducers [39]. This quorum sensing system regulates surface motility, biofilm formation, and pellicle formation in *A. baumannii* [39,50,51]. In *P. aeruginosa*, (p)ppGpp and DksA negatively regulate the transcription of autoinducer synthase genes, biosynthesis of autoinducers, and inhibit quorum sensing-dependent virulence factors [52,53]. However, this study showed that *dksA* is essential for the full expression of *abaI* and *abaR* and production of autoinducers in *A. baumannii* since the ∆*dksA* mutant exhibited reduced expression of *abaI* and *abaR*, and reduced production of autoinducers. Surface motility correlated with the production of autoinducers in ∆*dksA* mutants, whereas biofilm formation of ∆*dksA* mutant was increased compared with the WT strain. Our results suggest that DksA regulates the production of autoinducers through the expression of *abaI* and *abaR*. However, we cannot exclude the possibility that low production of autoinducers in the ∆*dksA* mutant results from growth retardation of this mutant strain in bioassay plates because the production of autoinducers depends on cell population density.

The roles of DksA in the pathogenesis of *A. baumannii* were assessed in an *in vivo* infection model. In this study, we used neutropenic mice because *A. baumannii* commonly infects severely ill patients or immunocompromised hosts [1]. CFUs in the lungs of surviving mice were significantly different between the mice infected with WT and ∆*dksA* mutant strains. Mice survival was also significantly different between WT and ∆*dksA* mutant strains. However, CFUs in the blood were not different between the two groups because of the big variation in CFUs in each experimental group and the low number of surviving mice. The ∆*dksA* mutant exhibited lesser virulence than the WT strain in the *in vivo* mouse model. The ∆*dksA* mutant exhibited growth retardation under static conditions, filamentous morphology, low expression of *ompA*, and low production of autoinducers *in vitro*, possibly contributing to low virulence of ∆*dksA* mutant in the *in vivo* infection model. Thus, DksA is a potential target for anti-virulence agents against *A. baumannii*.

This study identifies *dksA* in *A. baumannii. dksA* is required to maintain cellular morphology and growth under static conditions in *A. baumannii*. Furthermore, DksA is directly or indirectly involved in regulating genes associated with virulence factors, which contributes to the pathogenesis of *A. baumannii*. Overall, *A. baumannii* DksA plays multifaceted roles in bacterial physiology and virulence. Further studies are required to understand the regulatory mechanisms of multiple genes linked with virulence factors by DksA in *A. baumannii*.

## Supplementary Material

Supplemental Material

## Data Availability

The authors confirm that the data supporting the findings of this study are available within the article and its supplementary materials.
