# Supplementary material for: Global regulator DksA modulates virulence of Acinetobacter baumannii
Source: Virulence. 2021 Nov 9;12(1):2750–63. doi: 10.1080/21505594.2021.1995253 (PMC8583241; doi:10.1080/21505594.2021.1995253)
Supplement: Supplemental Material [file KVIR_A_1995253_SM3312.docx]

**Supplementary materials**

**Supplementary Table S1.** Bacterial strains and plasmids used in this study

| Bacteria/plasmids | Relevant characteristics^*^ | Reference of source |
| --- | --- | --- |
| *A. baumannii* |  |  |
| ATCC 17978 | Wild-type strain | ATCC |
| KM0248D | ∆*A1S_0248* of *A. baumannii* ATCC 17978 | This study |
| KM0248C | *A1S_0248* with T1 terminator in KM0248D | This study |
| *E. coli* |  |  |
| DH5α pir (Sy327 λ pir) | *endA1 hsdR17* *glnV44* (= *supE44*) *thi-1 recA1 gyrA96 relA1* φ80d*lac*Δ(*lacZ*)M15 Δ(*lacZYA-argF*)U169 *zdg-232::Tn10* *uidA::pir*+ | Laboratory collection |
| Plasmids |  |  |
| pDM4 | Suicide vector, *ori* R6K; Cm^R^; sacB | GenBank accession no. KC795686 |
| pOH4 | pHKD01 with *ompA* coding region of *A. baumannii* ATCC17978 under control of its native promoter with *nptI*; Km^R^ | [1] |

^*^Abbreviations. Cm^R^, chloramphenicol-resistant; Km^R^, kanamycin-resistant.

**Supplementary Table S2.** Primers used for the DNA cloning in this study

| Primer | Sequence (5`→3`)^*^ |
| --- | --- |
| glmS up_ SpeⅠ_F | GGACTAGTTGGTTTGAGCAATTGACTTGG |
| glmS up_R | GGGTTATATAAACTTTAGGGGCCTTAATAATGATCTTTTTTGAATTACTCTACAG |
| A1S_0248_F | GATCATTATTAAGGCCCCTAAAGTTTATATAACCCAAAAGAATTATGAC |
| A1S_0248_R | TTTTATTTGATGCCTTAACCGTTATTTTGCTTCTCTTTAATTTCTG |
| T1 terminator_F | AAATAACGGTTAAGGCATCAAATAAAACGAAAGGCTCA |
| T1 terminator_R | AGCAGGTGATTCTAGGGCGGCGGATTTG |
| glmS down_F | GCCGCCCTAGAATCACCTGCTTTAATAATTGATTGATTAAGC |
| A1S_0248_npt1_F | CCGAAGTGATGGTCTGCCTCGTGAAGAAGGTG |
| Npt1_SpeⅠ_R | GTTACTAGTGATCCGTCGACCTGCAGG |
| Npt1_ApaⅠ_R | GTTGGGCCCGATCCGTCGACCTGCAGG |
| glms up_seq_F | TGGCGAAGTCAGTAACTGTAGA |
| glms down_seq_R | GGAAAGCTTCTGTGTAGCGATATG |
| A1S_0248_630bp_F | CGTGAAGGCCCTAGTCTG |
| A1S_0248_630bp_R | ATGTAAAGGGCCGGTTGG |
| A1S_0248_UP_ApaⅠ_F | GTTGGGCCCCCGAAGAGGTAGGACGGTGAC |
| A1S_0248_UP_R | CTTAACCGTCACAATTACATAATAGGGCATTCCTCATCATACACGTATTATC |
| A1S_0248_down_F | GGAATGCCCTATTATGTAATTGTGACGGTTAAGAATTCTCCGGC |
| A1S_0248_down_R | GAGGCAGACCATCACTTCGGGACGATCTAAATCAACC |

^*^Underlined sequences indicate regions that are not complementary to the templates.

**Supplementary Table S3.** Primers used for qPCR in this study

| Primers | Sequence (5´ to 3´) | Target genes |
| --- | --- | --- |
| 16S rRNA-F | GCACAAGCGGTGGAGCAT | 16S rRNA |
| 16S rRNA-R | CGAAGGCACCAATCCATCTC |  |
| DksA-F | TCTTGGGTAGCACGGTCATTT | *dksA* |
| DksA-R | GGAAGGACAGCTCGAGCATT |  |
| BfmS-F | TTGAACTTATTCCACCGCCTTT | *bfmS* |
| BfmS-R | GCCCGTAATCCGAACTTTGTT |  |
| BfmR-F | GTTTAACCGTTTGTCGTG | *bfmR* |
| BfmR-R | GTGGTTGAACTGGTTTCG |  |
| CsuC-F | AAAGCAGGCGAGAAGCATATG | *csuC* |
| CsuC-R | GGATCGGCAACTCATCTACAATC |  |
| CsuD-F | ACCCTATCAAGGCGGTTCAAC | *csuD* |
| CsuD-R | CGACGATAGCCGTCATTATCTACA |  |
| CsuE-F | TCAGACCGGAGAAAAACTTAACG | *csuE* |
| CsuE-R | GCCGGAAGCCGTATGTAGAA |  |
| OmpA-F | TTGCACTTGCTACTATGCTTGTTG | *ompA* |
| OmpA-R | TGGCTGTCTTGGAAAGTGTAACC |  |
| AbaI-F | CTACTACCCACCACACAACCCTATTT | *abaI* |
| AbaI-R | GGTGAGCAGGGAATAGGCATT |  |
| AbaR-F | CGACCAAACAAGAGGTTGAAGTG | *abaR* |
| AbaR-R | GTCGGTTGGGCTCAGTCAA |  |

(A)


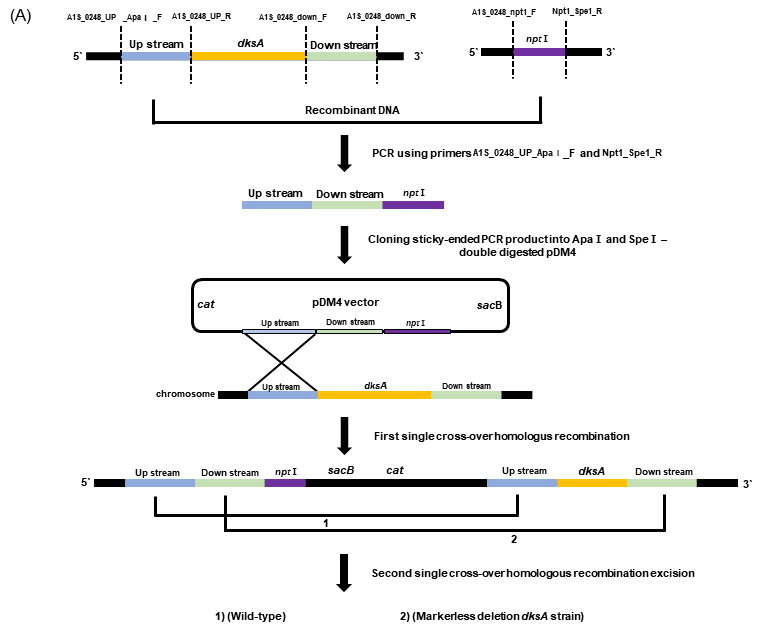


(B)


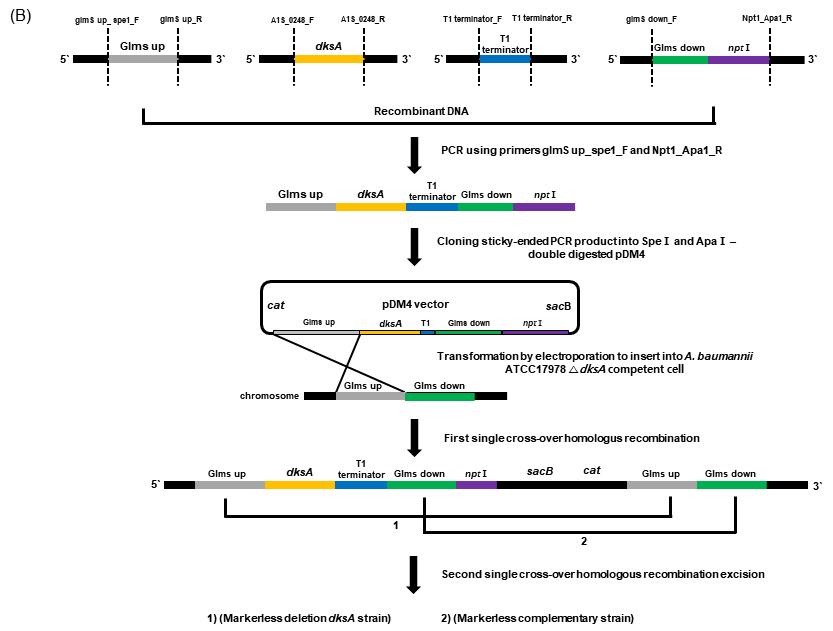


(C) (D)


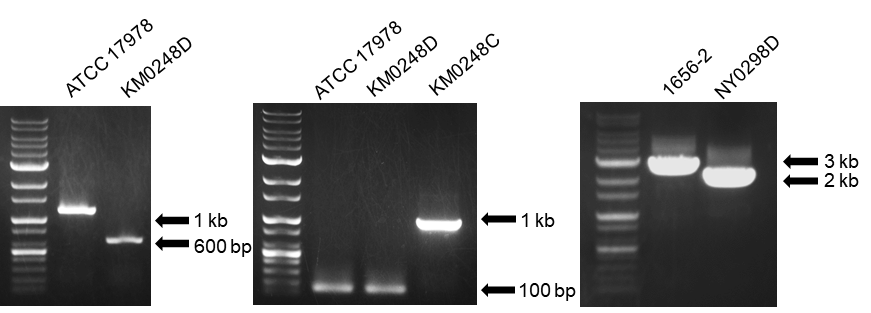

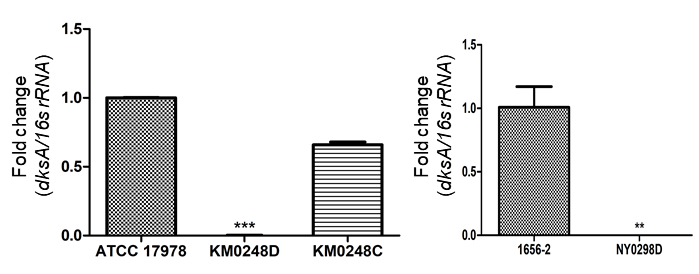


**Supplementary Fig. S1.** Construction of the ∆*dksA* mutant and *dksA*-complemented strains. (A) The construction of ∆*dksA* mutant (KM0248D) in *A. baumannii* ATCC 17978. (B) The construction of *dksA*-complemented strain (KM0248C) in KM0248D strain. (C) The deletion of *dksA* in KM0248D strain was confirmed by PCR using the primers A1S_0248_630bp_F and A1S_0248_630bp_R (Supplementary Table S2). The expected amplicon sizes, 1,161 bp and 630 bp, were identified in WT and KM0248D mutant strains, respectively. The complementation of *dksA* in KM0248C was confirmed by PCR using the primers glms up_seq_F and glms down_seq_R (Supplementary Table S2). The insertion of *dksA* in the KM0248C strain was identified by the amplicon size of 998 bp. (D) The expression of *dksA* was determined in the WT, ∆*dksA* mutant and *dksA*-complemented strains using qPCR. The data are presented as mean ± SD of three independent experiments. *** *p* <0.001 compared to WT strain.

**
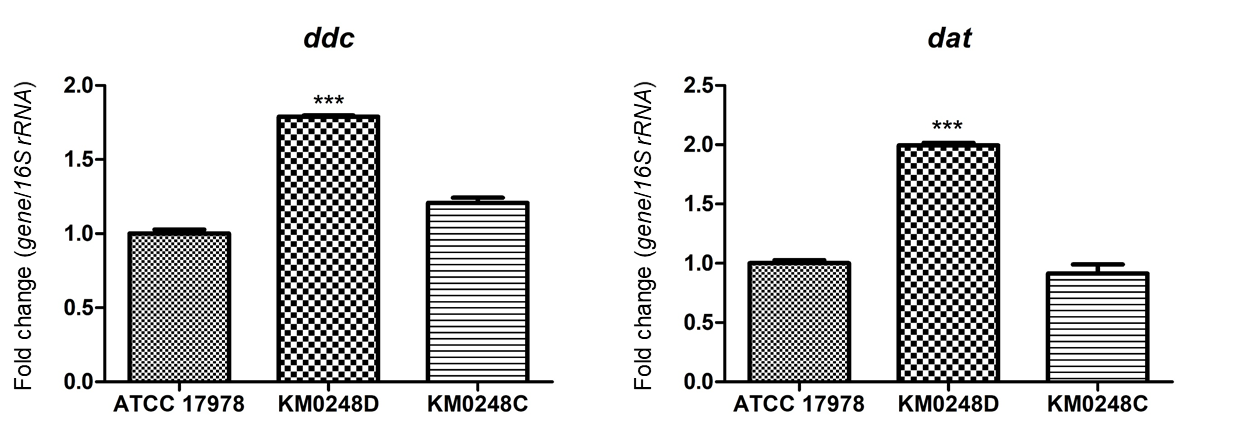
**

**Supplementary Fig. S2.** Expression of *ddc* and *dat* for 1,3-diaminopropane biosynthesis in *A. baumannii* strains. WT *A. baumannii* ATCC 17978, ∆*dksA* mutant (KM0248D), and *dksA*-complemented (KM0248C) strains were cultured in LB under static conditions for 12 h. Total RNA was extracted, and cDNA was synthesized. Gene expression was analyzed using qPCR. The data are the mean ± SD expression levels of the target genes in each strain relative to the expression of these genes in the WT strain. The experiments were performed three times independently. *** *p* <0.001 compared with the WT strain.

**Supplementary Materials and methods**

**Construction of the ∆*A1S_0248* mutant strain in *A. baumannii* ATCC 17978**

The *A1S_0248* gene of *A. baumannii* ATCC 17978 was deleted by a markerless gene deletion method (Supplementary Fig. S1A) [1]. The genomic DNAs purified from *A. baumannii* ATCC 17978 and pOH04 vector (Supplementary Table S1) for amplification of *A1S_0248* and kanamycin resistance cassette were used as PCR templates, respectively. The upstream and downstream regions of *A1S_0248* were combined with *nptI* by overlap extension PCR using specific primers (Supplementary Table S2). This DNA fragment was ligated into *Apa*I and *Spe*I-digested pDM4 (Supplementary Table S1). *E. coli* DH5α pir strain containing pDM4 which carried the mutated DNA fragment was used as a conjugal donor to *A. baumannii* ATCC 17978. Conjugation and isolation of the transconjugants were performed as previously described [2]. Deletion of *A1S_0248* in *A. baumannii* ATCC 17978 was confirmed by PCR analysis (Supplementary Fig. S1C) and the ∆*A1S_0248* mutant strain was named KM0248D (Supplementary Table S1).

**Complementation of *A1S_0248* in the KM0248D strain**

The *A1S_0248* coding region with its native promoter and T1 terminator was inserted into the downstream of *glmS* in the chromosome of *A. baumannii* ATCC 17978 using an overlap extension PCR (Supplementary Fig. S1B). A DNA fragment, in which *dksA* coding region with its native promoter, T1 terminator and the upstream and downstream regions of the insertion site were fused by overlap extension PCR using specific primers (Supplementary Table S2), was ligated into *Spe*I- and *Apa*I-digested pDM4 (Supplementary Table S1). This plasmid was integrated into the chromosome of the KM0248D mutant strain by transformation and homologous recombination. Insertion of the *A1S_0248* coding region with its native promoter and T1 terminator was confirmed by PCR analysis (Supplementary Fig. S1C). The *A1S_0248*-complemented strain was named KM0248C (Supplementary Table S1).

**References**

[1] Kwon H Il, Kim S, Oh MH, Na SH, Kim YJ, Jeon YH, et al. Outer membrane protein A contributes to antimicrobial resistance of *Acinetobacter baumannii* through the OmpA-like domain. J Antimicrob Chemother 2017;72:3012-5.

[2] Oh MH, Lee JC, Kim J, Choi CH, Han K. Simple method for markerless gene deletion in multidrug-resistant *Acinetobacter baumannii*. Appl Environ Microbiol 2015;81:3357-68.
